# Effect of Electron Irradiation Fluence on InP-Based High Electron Mobility Transistors

**DOI:** 10.3390/nano9070967

**Published:** 2019-07-01

**Authors:** Shuxiang Sun, Peng Ding, Zhi Jin, Yinghui Zhong, Yuxiao Li, Zhichao Wei

**Affiliations:** 1 School of Physics and Engineering, Zhengzhou University, Zhengzhou 450001, China; 2 Institute of Microelectronics, Chinese Academy of Sciences, Beijing 100029, China; 3 China Academy of Space Technology, Beijing 100086, China

**Keywords:** InP-based high electron mobility transistor, electron irradiation, DC and RF characteristics, kink effect

## Abstract

In this paper, the effect of electron irradiation fluence on direct current (DC) and radio frequency (RF) of InP-based high electron mobility transistors (HEMTs) was investigated comprehensively. The devices were exposed to a 1 MeV electron beam with varied irradiation fluences from 1 × 10^14^ cm^−2^, 1 × 10^15^ cm^−2^, to 1 × 10^16^ cm^−2^. Both the channel current and transconductance dramatically decreased as the irradiation fluence rose up to 1 × 10^16^ cm^−2^, whereas the specific channel on-resistance (*R*_on_) exhibited an apparent increasing trend. These changes could be responsible for the reduction of mobility in the channel by the irradiation-induced trap charges. However, the kink effect became weaker with the increase of the electron fluence. Additionally, the current gain cut-off frequency (*f*_T_) and maximum oscillation frequency (*f*_max_) demonstrated a slightly downward trend as the irradiation fluence rose up to 1 × 10^16^ cm^−2^. The degradation of frequency properties was mainly due to the increase of gate-drain capacitance (C_GD_) and the ratio of gate-drain capacitance and gate-source capacitance (C_GD_/C_GS_). Moreover, the increase of *R*_on_ may be another important factor for *f*_max_ reduction.

## 1. Introduction

With the development of high-precision detection and high-speed data transmission applications, the operating frequency of the core chips in transceiver systems has increased from W band to G band and even terahertz [1,2,3]. Confined to physical constraint and manufacturing cost, the typical channel length of metal–oxide–semiconductor field-effect transistors (MOSFETs) is hard to downscale further after the 10 nm node. For nearly several decades, III-V compound semiconductor transistors have stood out and become competitive alternatives for devices based on either advanced micro/nano-fabrication technologies or novel structures [4,5,6]. Benefiting from electron beam lithography (EBL) and molecular beam epitaxy (MBE) techniques, the current gain cut-off frequency (*f*_T_) and maximum oscillation frequency (*f*_max_) of InP-based high electron mobility transistors (HEMTs) have been reported to be over 700 GHz [7] and 1 THz [8], respectively. Therefore, InP-based HEMTs are potentially excellent candidates for various millimeter-wave circuits of transceiver systems in space applications, such as national defense, aerospace, and satellite radar [9].

Radiation tolerance is critical for semiconductor devices which operate in space missions [10]. In the space environment, various high-energy particles and rays damage electronic devices, resulting in performance degradation of the devices and even the abnormality of electronic systems [11,12]. Admittedly, the irradiation damage mechanism of various devices with III-V materials have been widely reported [13,14,15,16,17,18]. Oh et al. studied the effects of electron irradiation on the gate leakage current of AlGaN/GaN HEMTs. The results showed that the gate leakage current significantly decreased, which was due to the neutralization of nitrogen vacancies, and that the removal of oxygen impurities induced the defects after electron irradiation [14]. Kimura et al. performed a systematic study of electron irradiation effects on the Schottky gate of InGaAs/GaAs HEMTs which showed that deep traps were introduced at the gate metal and GaAs layer [15]. The impact of proton irradiation on the direct current (DC) performance of AlGaN/GaN HEMTs was investigated by Liu et al., which indicated that the DC characteristics were degraded more seriously at high proton fluence. This was due to the greater reduction of the electron concentration and mobility at high proton fluence [17]. Most of these research studies are focused mainly on GaN and GaAs materials. However, only a few articles have investigated the irradiation effects on InP-based HEMT devices, especially the effects on the Kink effect and radio frequency (RF) characteristics. Actually, electrons are major radiation particles in the low earth orbit, where many satellites and space stations are found. Incident electrons create lattice defects in semiconductor devices, such as vacancies, interstitials, and complex defects, which are induced by atomic displacements [19]. Moreover, electron irradiation can also generate trapped charges in the dielectrics, such as the gate oxide layer [20,21]. These defects that act as carrier recombination centers or trapping centers are bound to cause a decrease in the DC and RF characteristics of devices. Therefore, it is essential to explore the impact of electron irradiation on InP-based HEMTs.

In this article, 1 MeV electron irradiation was carried out on InP-based HEMTs at fluences ranging from 1 × 10^14^ cm^−2^, 1 × 10^15^ cm^−2^, to 1 × 10^16^ cm^−2^. On this basis, the changes and damage mechanism of direct current (DC) and radio frequency (RF) properties were analyzed before and after electron irradiation. These studies serve as a theoretical underpinning for radiation-harden design of devices and integrated circuits, further improving the stability and durability of related electronic systems.

## 2. Materials and Methods

**Figure 1:**
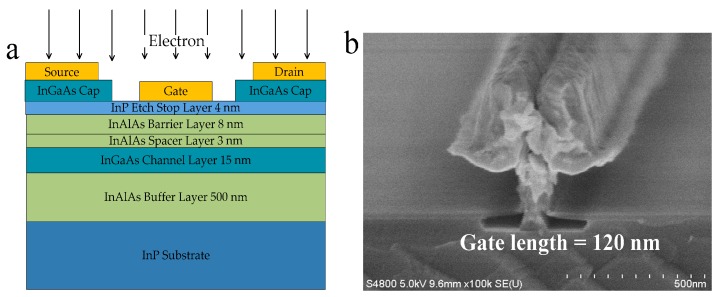
(**a**) Epitaxial layer structure of the InP-based high electron mobility transistor (HEMT), (**b**) scanning electron microscope (SEM) photographs of a vertical profile of the T-gate of InP-based HEMTs.

The InAlAs/InGaAs InP-based HEMTs were developed on a 4-inch compound semiconductor manufacturing assembly line at the Institute of Microelectronics, Chinese Academy of Sciences. The epitaxial structures were successively grown by gas source molecular beam epitaxy (GSMBE) with parameters as shown in Figure 1a. The entire epilayers from bottom to top consisted of an InAlAs buffer, an InGaAs channel, an unstrained InAlAs spacer layer, a Si-doped plane, an lnAlAs Schottky barrier layer, an InP etching stopper layer, and uppermost composite InGaAs cap layers. The composite cap layers were adopted to improve contact characteristics, which contained a highly Si-doped In_0.6_Ga_0.4_As cap layer (3 × 10^19^ cm^−2^) and a Si-doped In_0.53_Ga_0.47_As transition layer (5 × 10^18^ cm^−2^). All InAlAs layers were lattice matched with the InP substrate.

The InP-based HEMTs’ fabrication process began with mesa isolation through wet chemical etching based on phosphorus acid, leaving an In_0.52_Al_0.48_As buffer layer exposed on the surface. Then, ohmic contacts of source and drain electrodes were formed using non-alloyed Ti/Pt/Au with a 2 μm separation. A 20 nm thick SiO_2_ was deposited to improve resist adhesion, passivate the device surface, and mechanically support the T-shaped gate. Subsequently, Ti/Au connection wires were formed as coplanar waveguide bond pads. Afterward, 120 nm T-shaped gates were patterned using the EBL technique, and the fine gate-foot pattern was precisely replicated on the SiO_2_ film. Subsequently, Ti/Pt/Au metallization was carried out and followed by gate-recess etching until the InP etching-stopper layer. Figure 1b depicts a detailed vertical profile of a T-gate by scanning electron microscope (SEM). The detailed fabrication process has also been mentioned elsewhere in [22,23].

InP-based HEMTs with 2 × 50 μm gate width and 120 nm gate length were then irradiated by 1 MeV electrons with fluences of 1 × 10^14^ cm^−2^, 1 × 10^15^ cm^−2^, and 1 × 10^16^ cm^−2^ at room temperature at the Lanzhou Institute of Physics of China. In particular, one quarter of a device wafer from a set of the complete technological process was carved into small pieces for experiments with different fluences, and the average value of three devices was determined for each fluence irradiation to improve precision. The DC and RF characteristics of these devices were measured within 12 h by using a B1500A semiconductor parameter analyzer and N5245A PNA-X vector network analyzer (Keysight Technologies, Made in Malaysia). All the measurements were carried out at room temperature.

## 3. Results and Discussion

**Figure 2:**
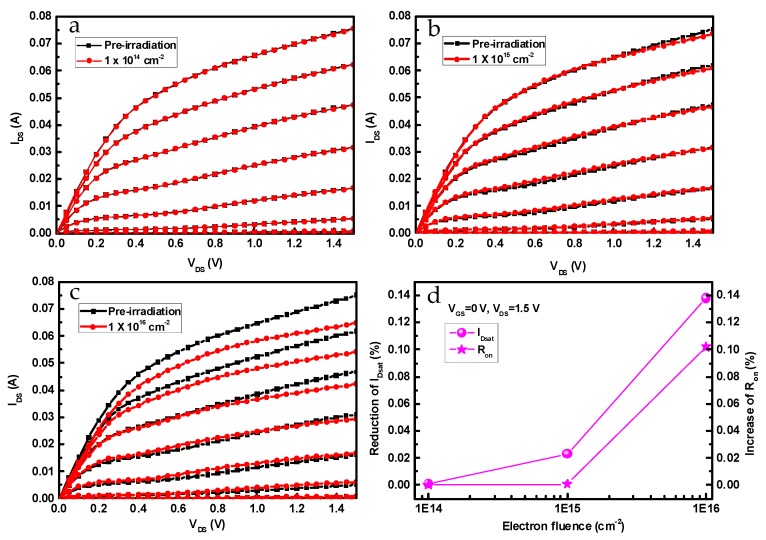
Output characteristics of InP-based HEMTs before and after 1 MeV electron irradiation with different irradiation fluences. (**a**) 1 × 10^14^ cm^−2^, (**b**) 1 × 10^15^ cm^−2^, (**c**) 1 × 10^16^ cm^−2^, (**d**) *I*_D,sat_ and *R*_on_ at *V*_GS_ = 0 V.

**Figure 3:**
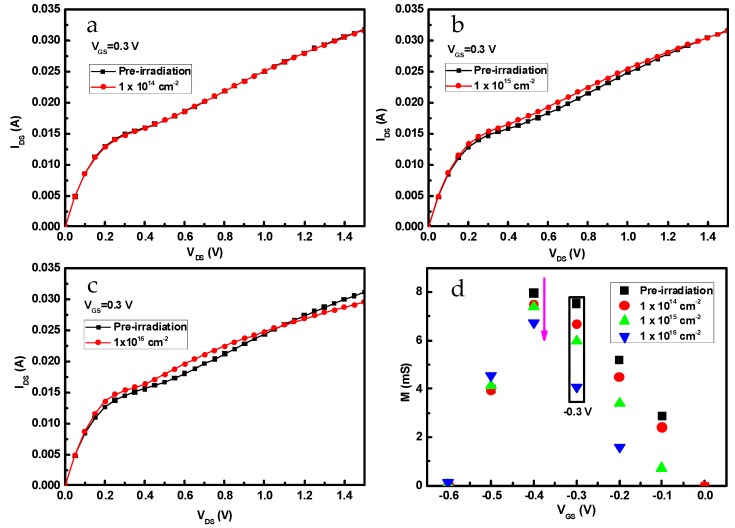
Output characteristics of InP-based HEMTs before and after 1 MeV electron irradiation with *V*_GS_ = −0.3 V. (**a**) 1 × 10^14^ cm^−2^, (**b**) 1 × 10^15^ cm^−2^, (**c**) 1 × 10^16^ cm^−2^, (**d**) M impact factor.

Figure 2a–c indicates the output characteristics of InP-based HEMTs before and after 1 MeV electron irradiation with fluences of 1 × 10^14^ cm^−2^, 1 × 10^15^ cm^−2^, and 1 × 10^16^ cm^−2^. Gate-source voltage (*V*_GS_) varies positively from −0.6 V to 0 V in steps of 0.1 V, whereas drain-source voltage (*V*_DS_) sweeps from 0 V to 1.5 V in steps of 0.05 V. It has been found that channel current is nearly unchanged until irradiation fluence reaches 1 × 10^15^ cm^−2^; afterward, the channel current (*I*_DS_) shows a dramatic drop as irradiation fluence rises. Specifically, saturation channel current (*I*_D__,sat_) and specific channel on-resistance (*R*_on_) are extracted from the output curves at *V*_GS_ = 0 V, as shown in Figure 2d. With irradiation fluence increasing to 1 × 10^16^ cm^−2^, *I*_D,sat_ and *R*_on_ have deteriorated by 13.8% and 10.2%, respectively. From the output characteristics, the drain-source current was abruptly increased at the lower gate bias, which was the so-called kink effect, as shown in Figure 2. Figure 3 shows the *I*_DS_-*V*_DS_ curve at *V*_DS_ = −0.3 V. The results indicate that the kink effect was alleviated with the increase of electron fluence, as shown in Figure 3a–c. To understand the variation of the kink effect, the M impact factors with *V*_GS_ at different electron fluences were calculated, as shown in Figure 3d. With the increase of electron fluence, the M impact factor cuts down and the kink effect becomes weaker. This may be owing to the reduction of the surface states at the gate-recess region after electron irradiation [14].

**Figure 4:**
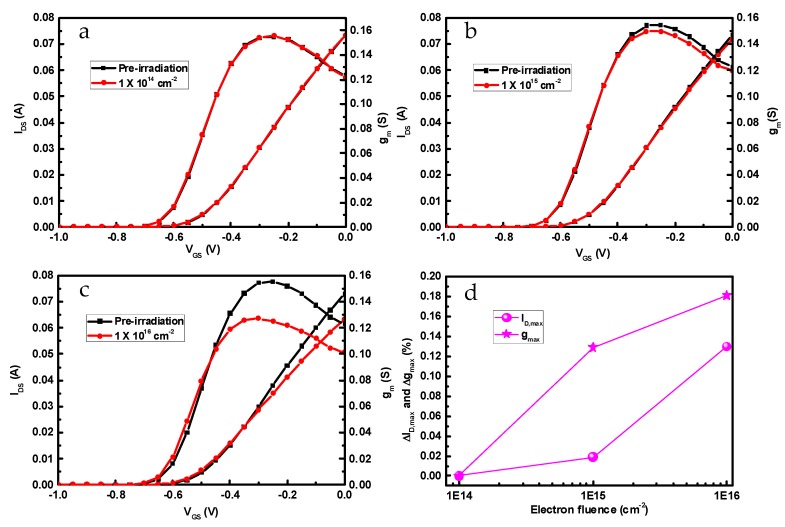
Transfer characteristics of InP-based HEMTs before and after 1 MeV electron irradiation with different irradiation fluences. (**a**) 1 × 10^14^ cm^−2^, (**b**) 1 × 10^15^ cm^−2^, (**c**) 1 × 10^16^ cm^−2^, (**d**) Δ*g*_m,max_ and Δ*I*_D,max_.

Figure 4 describes the transfer characteristics of InP-based HEMTs before and after 1 MeV electron irradiation with *V*_DS_ = 1.4 V. From the transfer curves, the transconductance (*g*_m_) and channel current (*I*_DS_) were observed to decrease after an irradiation fluence larger than 1 × 10^16^ cm^−2^, and the reduction rates (Δ) were computed precisely and are shown in Figure 4d. The maximum transconductance (*g*_m,max_) is slumped by 18.1% and the maximum channel current (*I*_D,max_) drops down by 12.9% at an electron fluence of 1 × 10^16^ cm^−2^.

**Figure 5:**
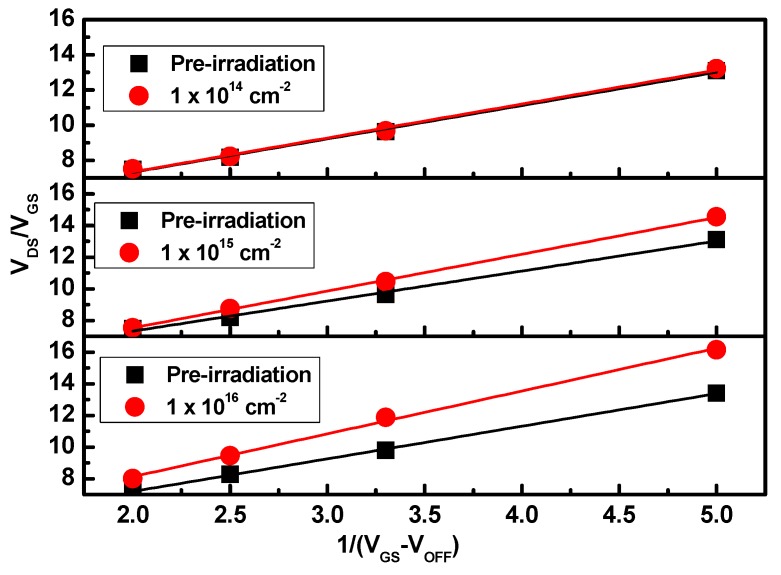
The variation *V*_DS_/*I*_DS_ as a function of 1/(*V*_*GS*_ − *V*_OFF_) for different electron fluences.

**Figure 6:**
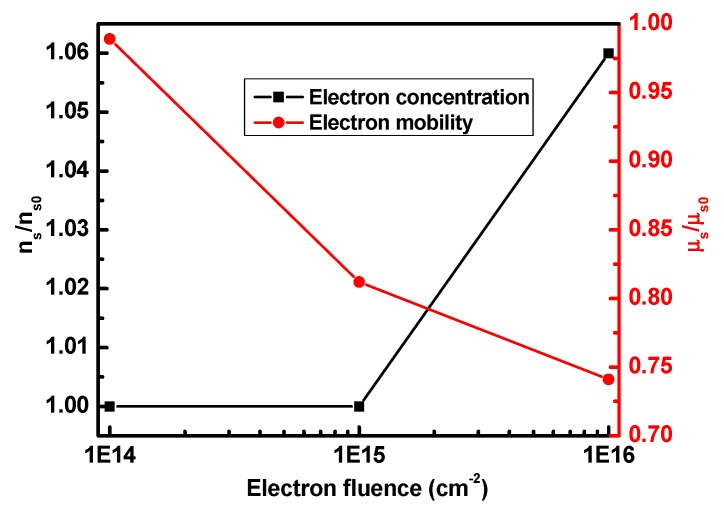
The variation of electron concentration and mobility with the electron fluence.

For HEMT devices, the primary factors which contribute to the change of DC characteristics are the carrier density and mobility in the hetero-junction region. Charged defects in the hetero-junction region are induced by electron irradiation. They may act as compensation centers of majority carriers in the channel region, that is, the carrier removal effect [24]. Meanwhile, the mobility of carriers in the channel changes when their trajectories are affected by irradiation-induced charged defects [25]. To understand the irradiation-induced charged defects on the carrier density and carrier mobility, a charge control model was used, and it can be written as follows [26]:

(1) $$n_{s}=\frac{\varepsilon }{qd}\left(V_{\mathrm{GS}}-V_{OFF}\right),\;\frac{V_{\mathrm{DS}}}{I_{\mathrm{DS}}}=R_{S}+R_{D}+\frac{Ld}{\mu W\varepsilon \left(V_{\mathrm{GS}}-V_{\mathrm{OFF}}\right)},$$

where *V*_OFF_ is the off voltage, *R*_S_ and *R*_D_ are the source and drain access resistance, L is the gate length, and W is the gate width. The carrier mobility can be obtained from the slope of the *V*_DS_/*I*_DS_ − 1/(*V*_GS_ − *V*_OFF_) curves, as shown in Figure 5. Figure 6 shows the variation of electron concentration and mobility with the electron fluence. The results show that the electron concentration increases slightly and the electron mobility reduces by 25.9% at the electron fluence of 1 × 10^16^ cm^−2^. This may be due to the fact that some incident particles may remain in the device structure apart from inducing a reasonable number of defect states [27]. The particles that remained and the induced defects result in the decrease of carrier mobility by scattering. Meanwhile, the incident particles may rearrange the mobile defect states, which lead to some electrons being released from defect sites, and finally the carrier density increases slightly with electron fluence. Therefore, the degeneration of device characteristics are mainly due to the decreasing carrier mobility by electron irradiation.

As a kind of high frequency device, *f*_max_ affects the device power gain of the analog circuit, while *f*_T_ determines the switching speed of the digital circuit. The S-parameters of the InP-based HEMTs irradiated by different fluences were measured over frequencies from 0.1 to 40 GHz with a step size of 0.1 GHz. The parasitic capacitance and inductance induced by pad metals were calibrated and subtracted through open and short pads on the same wafer. The value of *f*_T_ and *f*_max_ can be respectively extrapolated from the current gain (H_21_) and maximum available/stable power gain (MAG/MSG) using a least squares fitting with a −20 dB/decade slope. The *f*_T_ and *f*_max_ can be written as follows [28]:

(2) $$f_{T}=\frac{g_{m}}{2\pi \left(C_{\mathrm{gd}}+C_{\mathrm{gs}}\right)}$$

(3) $$f_{\max}=\frac{f_{T}}{\sqrt{4g_{d}\left(R_{S}+R_{i}+R_{G}\right)+2\left(C_{\mathrm{GD}}/C_{\mathrm{GS}}\right)\left[\left(C_{\mathrm{GD}}/C_{\mathrm{GS}}\right)+g_{m}\left(R_{S}+R_{i}\right)\right]}}$$

where *g*_d_ are the out conductance, *R*_G_, *R*_S_ and *R*_i_ are the gate, source and channel resistance, respectively, and *C*_GS_ and *C*_GD_ are the gate-source capacitance and gate-drain capacitance.

**Figure 7:**
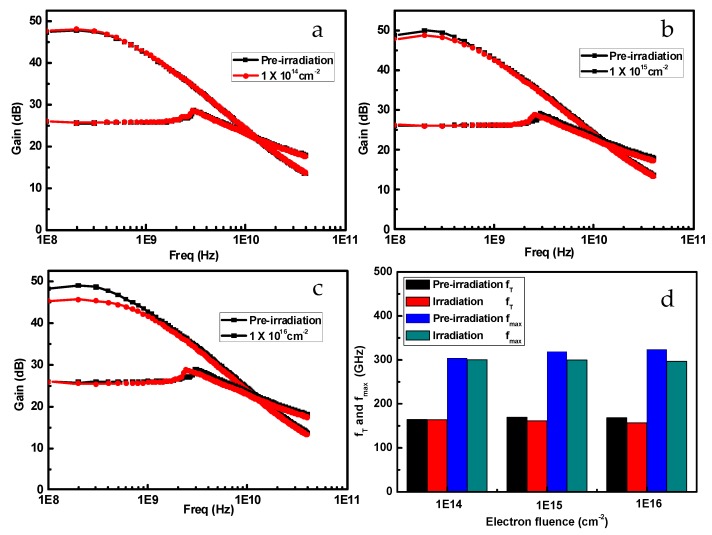
Frequency characteristics of InP-based HEMTs before and after 1 MeV electron irradiation with different irradiation fluences. (**a**) 1 × 10^14^ cm^−2^, (**b**) 1 × 10^15^ cm^−2^, (**c**) 1 × 10^16^ cm^−2^, (**d**) variation of *f*_T_ and *f*_max_ with different electron fluences.

**Figure 8:**
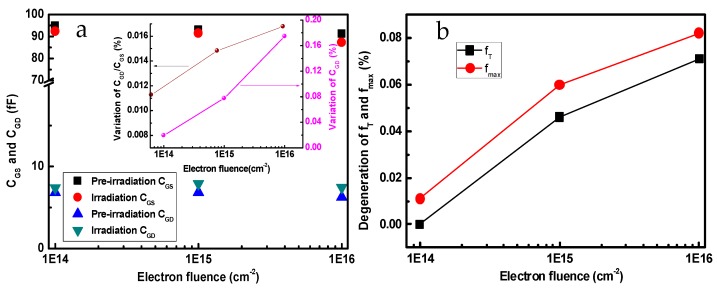
(**a**) Dependence of *C*_GS_ and *C*_GD_ on electron fluences, (**b**) degradation percentage of *f*_T_ and *f*_max_ as a function of electron fluence.

Figure 7 shows the frequency characteristics of InP-based HEMTs before and after 1 MeV electron irradiation with different electron fluences. With the increase of the fluence, both *f*_T_ and *f*_max_ demonstrate a slightly downward trend. From Equations (1) and (2), *C*_GS_, *C*_GD_, and *C*_GD_/*C*_GS_ are the main factors impacting the *f*_T_ and *f*_max_ of devices. Figure 8a show the variation of *C*_GS_ and *C*_GD_ as a function of electron fluence. The results show that the C_GD_ is increasing as the electron fluence rises for the samples. For larger electron fluences, the values of *C*_GD_/*C*_GS_ become larger than before, as shown in the inset of Figure 8a. However, *C*_GS_ decreases as the electron fluence increases. From Equations (1) and (2), the increase of *C*_GD_ and *C*_GD_/*C*_GS_ are the main reason for the degradation of the *f*_max_ and *f*_T_ values. In addition, electron irradiation significantly reduces the carrier concentration in the channel and results in an increase of *R*_on_, which may be another important factor for *f*_max_ reduction. Figure 8b shows the degradation percentage of *f*_T_ and *f*_max_ in the case of different electron fluences. The *f*_T_ and *f*_max_ are only reduced by 7.1% and 8.2%, respectively, even when the electron fluence rose up to 1 × 10^16^ cm^−2^. Therefore, the InP-based HEMT device demonstrates good capability when it works in an irradiation environment.

## 4. Conclusions

In conclusion, the DC and RF characteristics of InP-based HEMTs were studied before and after 1 MeV electron irradiation. The degradation of DC properties became gradually apparent with the increase of electron fluence, which could attributed to the reduction of carrier mobility in the channel. Furthermore, *f*_T_ and *f*_max_ demonstrated a slightly downward trend. The deterioration of frequency properties was mainly due to the increase of *C*_GD_ with induced charged traps. Additionally, the specific channel on-resistance *R*_on_ increased after electron irradiation, which led to the reduction of *f*_max_. However, the *f*_max_ showed a degeneration of only 8.2%, even when the electron fluence rose up to 1 × 10^16^ cm^−2^. This shows that an InP-based HEMT device is perfectly suited to work in an irradiation environment.
